# Reply

**Published:** 1860-03

**Authors:** L. H. Hendrick


### REPLY.

BY L. H. HENDRICK, M. D., D. D. S.

Honored Sir, and respected Professors: In accepting your farewell greeting as a retiring class of students, and in receiving your right hand of fellowship, cordially welcoming this graduating class into the opening door of the honorable profession of Dental Surgery, I speak the language of the hearts of my associates when I say, Thanks, honored Faculty, for the memorable words of counsel you have just addressed us, and for that consistent course of admonition and instruction, which like the gentle guardians' care, have been nursing and nurturing us for transplantation into the world's field, for the bearing of fruit.

As true sons of the Ohio Dental College, in obedience to the wisest philosophy in which we have been instructed, I shall not occupy this consecrated hour in extended remarks, for we, as a class, subscribe to the sentiment. "Do much, say little, hear much, reflect upon the counsel of superiors; distrust our own opinions, and value the advice of the experienced." We are of your family and kindred; the initials of the profession, we trust, have been worthily attached to our names, for one and all we are going forth to the discharge of our duties, impressed with a deep sense of reverence for our instructors and for our Alma Mater.

We, however, have made up our minds to go into this "terra incognita" of the professional world with an exuberant flow of spirits. We see how successful our seniors have been, we know what youth can attain, we feel emulous to take a part in the tug of life, not as a holiday sport, but as a grand reality. We have got the clue from you; we have noted the finger-board guide you have set up, and are inspired by your example, to be students in the arts and mysteries of meeting the demands of the hour in the battle of life.

We have a professional pride to take your parchments into circles where a good name can be attained and maintained.

We shake your hand at parting, but we advise you that the thrill of sympathy—the contact of palms has betokened, will enliven each of your pupils to an energy corresponding to our natural gifts, our culture and the whispering monitor of college remembrance, and incite us to win our way to positions that are reserved for the new classes of graduates of this Dental College. We hope to honor in our words and actions our Alma Mater.

We have been told by D'Israeli that the history of heroes is the history of youth ; that the greatest captains of ancient times both conquered Italy at five and twenty ; that the work of the world has been done mainly by young brains. We know what the claims of the west are on all her citizens ; we appreciate what the spirit of the age demands of the professional man, and we ask here on the threshold of the College door, while looking around on the associates we leave and back with affection to our sage instructors, to say, that, in catching a glimpse through the opening door of duty, we pledge you, one and all, Professors of the Ohio College of Dental Surgery, that we will devote our whole energies to expand into active life the power you have aided in developing, and will with our own students, and by all other means honor and aid the noble institution whose zeal we have attesting our bachelor degree in Dental science. We love our profession and will avail ourselves of the practical advice you have given as to our relation and communications with our patrons. We speak confidently of having abundance of these, for we intend to deserve them.

Fellow-Students, of the graduating class, to you I know not what to say ; but five short months ago we met, many of us as strangers, within these walls as students, that our minds might be furnished with the acquisitions needful to an honorable prosecution of the practice of Dental Surgery ; hand in hand we have gone together through the arduous, anxious period of our pupilage, and to-night our friends have been called together to witness the receiving of our rewards, in the conferring the highest honors of a profession that deservedly ranks in usefulness, dignity and responsibility, with the most ennobling of the arts cultivated by or practiced among men.

And now, my beloved class-mates, here we are, in the presence of our instructors and the friends of the College; here our eyes are to behold for the last time the present scene. Eye to eye, no more are we to behold each other's lineaments, and when we grasp each others hands and tear ourselves from each other's embrace, we will experience that which is the heart's business, an experience designed for our instruction, for our humiliation and exaltation. Prudence, kindly, has placed us for a while in competition, apposition, and opposition. We know each other's strength; we know our mutual faults; we have been schooled in the higher branches of the knowledge of man, and now are crystalizing into tear-drops, the concentration of feelings which struggle at our hearts—we have met and learned together, we now part to learn and act apart—we bear imprinted on our hearts each other's image, the lovable and good survives—we look now on each other as at other parts of ourselves, going out into diverse parts of the world, but all the while being of one body. Here, too, the Dental College turns on a single pivot, the shaft on which our several pulleys are attached.

Brethren and class-mates, let us be brethren and class-mates in heart wherever dispersed. Let us be good men and good dentists. Let us honor our instructors and be linked in our destiny with the welfare of our beloved college.

"Let us boldly strive and win,—
Ours the work to plan and do
Till the nations gathered in,
Claim the race and victory too.

"Ever looking upward higher,
Nerve the soul for any strife;
To the distant goal aspire
Ne'er give over while there's life.

"Let us nobly strive to win,
Nobly plan and nobly do;
Till the harvest gathered in,
Ours the crown and victory too."

Fellow-student, the class now parting with you passes into the practical department, and you are left with our instructors in the theoretical department. Your morning salutations, your cheerful looks, your generous acts are to be hereafter for others. We cherish remembrance of you, and take the step that you are preparing to take—advance couriers, we herald your coming, and while clearing many snags and prongs out of the way before you, we hope to welcome you with your budget of improved formulas and patterns, into the great laboratory of life, where we trust you will find us up to our eyes in work, in model offices, in neat and tasty array, making our customers happy and ourselves worthily rich.

Worthy Professors—fathers in the profession, we give you our adieus—we honor you now, we hope to give you honor in the honor we hope to attain in our practice. Farewell, we go to further study, to fields of research, in which your instructions have fitted us to engage. May you live long in peace and prosperity. May your mid-day be unclouded, and your evening calm. May the hand of destiny deal gently with you. May the just rewards of science and virtue weave a chaplet for your brows, bright and unfading as the beams of light. May heaven bless the schemes of honest enterprise in which you have enlisted, and may you reap the plentiful harvest of honor, fame and emolument which you so richly deserve. Class-Mates, Fellow-Students, Professors—farewell.

"O! who that from his friends must sever
For long, long years, perhaps forever,
Would wish to fly without possessing
A parting look, a parting blessing;
Though in that moment is combined
All that can agonize the mind;

Though lips can not express their love,
Though tears may then refuse to flow;
Though anguish not to be expressed
Nearly o'er whelms the throbing breast?
Yet when the trying hours are o'er,
And friendly forms are seen no more—
When one fond look in view is sought
There's consolation in the thought
That the last look, the parting sigh,
Recorded their fidelity.
The silent pressure of the hand,
Which friends so well can understand—
The words, the looks, which though they fill
Our eyes with tears, are comfort still."

